# Supplementary material for: SARS-CoV-2 PCR cycle threshold at hospital admission associated with patient mortality
Source: PLoS One. 2020 Dec 31;15(12):e0244777. doi: 10.1371/journal.pone.0244777 (PMC7774957; doi:10.1371/journal.pone.0244777)

**S1 Fig. (Left Panel)**: Barchart of the proportions of missing values. (**Right Panel)**: all existing combinations of missing values (Red) and non-missing values (Blue) with the pattern frequency represented by small horizontal bars with the number involved indicated.


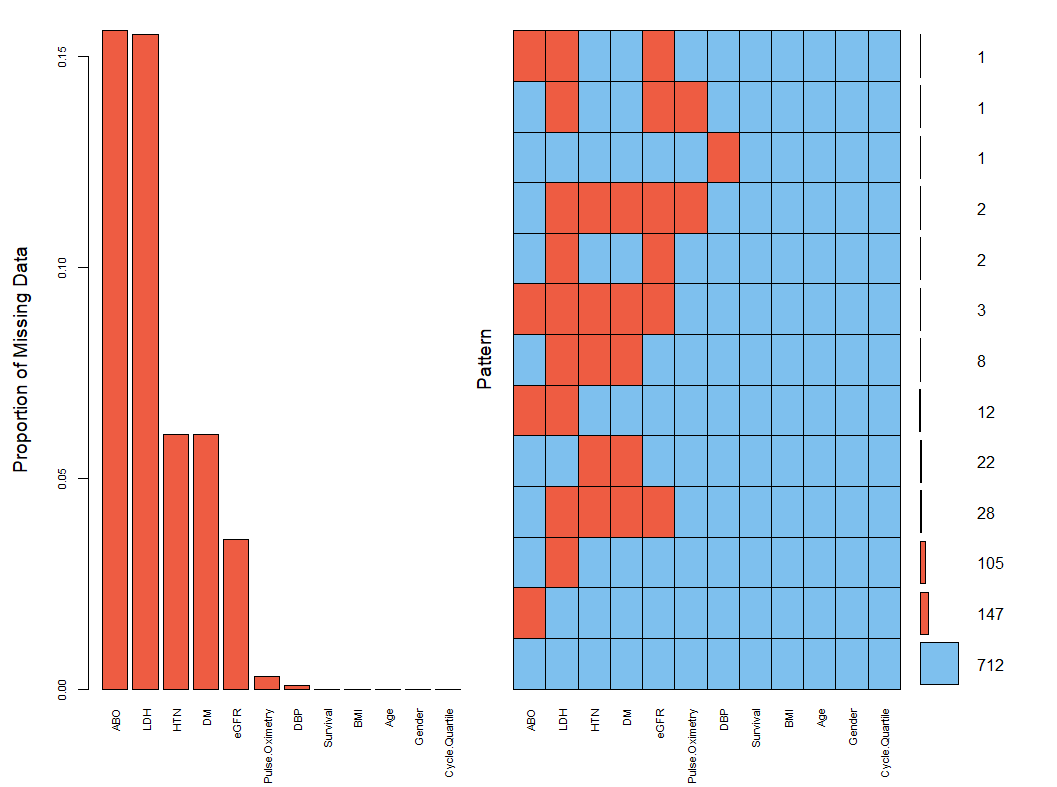

Supplement: S1 Fig — (Left Panel): Bar chart of the proportions of missing values. (Right Panel): all existing combinations of missing values (Red) and non-missing values (Blue) with the pattern frequency represented by small horizontal bars with the number involved indicated. (DOCX) [file pone.0244777.s001.docx]
