# Supplementary material for: SARS-CoV-2 PCR cycle threshold at hospital admission associated with patient mortality
Source: PLoS One. 2020 Dec 31;15(12):e0244777. doi: 10.1371/journal.pone.0244777 (PMC7774957; doi:10.1371/journal.pone.0244777)
Supplement: S1 Table — Time period has been divided into quarters based on PCR testing totals. (DOCX) [file pone.0244777.s002.docx]

**S1 Table.** Sensitivity Analysis: examining the effect of selected time period on model results. Time period has been divided into quarters based on PCR testing totals

|  | **Period 1** | | | **Period 2** | | | **Period 3** | | | **Period 4** | | |
| --- | --- | --- | --- | --- | --- | --- | --- | --- | --- | --- | --- | --- |
| ***Predictors*** | ***Odds Ratios*** | ***CI*** | ***p*** | ***Odds Ratios*** | ***CI*** | ***p*** | ***Odds Ratios*** | ***CI*** | ***p*** | ***Odds Ratios*** | ***CI*** | ***p*** |
| **(Intercept)** | 0.13 | 0.01 – 1.60 | 0.111 | 0.04 | 0.00 – 0.81 | **0.036** | 0 | 0.00 – 0.05 | **<0.001** | 0.04 | 0.00 – 0.70 | **0.028** |
| **Age** | 1.03 | 1.01 – 1.05 | **0.003** | 1.05 | 1.02 – 1.08 | **<0.001** | 1.08 | 1.05 – 1.11 | **<0.001** | 1.06 | 1.03 – 1.09 | **<0.001** |
| **Gender [M]** | 1.18 | 0.67 – 2.10 | 0.568 | 2.67 | 1.36 – 5.26 | **0.005** | 2.55 | 1.30 – 5.00 | **0.006** | 1.71 | 0.89 – 3.30 | 0.108 |
| **BMI** | 1.01 | 0.97 – 1.05 | 0.713 | 1.04 | 0.99 – 1.09 | 0.12 | 1.08 | 1.03 – 1.14 | **0.003** | 1.03 | 0.99 – 1.07 | 0.102 |
| **Cycle Threshold** | 0.95 | 0.90 – 1.00 | 0.053 | 0.9 | 0.85 – 0.95 | **<0.001** | 0.91 | 0.86 – 0.96 | **0.001** | 0.9 | 0.85 – 0.94 | **<0.001** |
| **Observations** | 261 | | | 261 | | | 261 | | | 261 | | |
