# Supplementary material for: SARS-CoV-2 PCR cycle threshold at hospital admission associated with patient mortality
Source: PLoS One. 2020 Dec 31;15(12):e0244777. doi: 10.1371/journal.pone.0244777 (PMC7774957; doi:10.1371/journal.pone.0244777)
Supplement: S2 Table — (DOCX) [file pone.0244777.s003.docx]

**S2 Table.** Sensitivity Analysis: examining the effect of covariate selection on study findings

|  | **Model 1** | | | **Model 2** | | | **Model 3** | | | **Model 4** | | | **Model 5** | | |
| --- | --- | --- | --- | --- | --- | --- | --- | --- | --- | --- | --- | --- | --- | --- | --- |
| ***Predictors*** | ***OR*** | ***CI*** | ***p*** | ***OR*** | ***CI*** | ***p*** | ***OR*** | ***CI*** | ***p*** | ***OR*** | ***CI*** | ***p*** | ***OR*** | ***CI*** | ***p*** |
| **Cycle Threshold** | 0.91 | 0.87 – 0.95 | **<0.001** | 0.91 | 0.88 – 0.95 | **<0.001** | 0.9 | 0.87 – 0.94 | **<0.001** | 0.92 | 0.89 – 0.95 | **<0.001** | 0.91 | 0.89 – 0.94 | **<0.001** |
| **Age** | 1.04 | 1.01 – 1.06 | **0.002** | 1.04 | 1.02 – 1.06 | **<0.001** | 1.05 | 1.03 – 1.07 | **<0.001** | 1.04 | 1.02 – 1.05 | **<0.001** | 1.03 | 1.02 – 1.05 | **<0.001** |
| **Gender [M]** | 2.26 | 1.30 – 3.94 | **0.004** | 2.01 | 1.27 – 3.20 | **0.003** | 2.08 | 1.34 – 3.23 | **0.001** | 2.08 | 1.40 – 3.09 | **<0.001** | 1.92 | 1.33 – 2.79 | **0.001** |
| **BMI** | 1.03 | 0.99 – 1.07 | 0.141 | 1.02 | 0.99 – 1.06 | 0.187 | 1.03 | 1.00 – 1.06 | 0.073 | 1.04 | 1.01 – 1.07 | **0.018** | 1.03 | 1.00 – 1.05 | 0.062 |
| **Pulse Ox** | 0.94 | 0.90 – 0.98 | **0.003** | 0.94 | 0.90 – 0.97 | **0.001** | 0.92 | 0.89 – 0.96 | **<0.001** | 0.9 | 0.87 – 0.93 | **<0.001** | 0.9 | 0.87 – 0.93 | **<0.001** |
| **DBP** | 0.94 | 0.92 – 0.96 | **<0.001** | 0.93 | 0.92 – 0.95 | **<0.001** | 0.94 | 0.92 – 0.95 | **<0.001** | 0.94 | 0.93 – 0.96 | **<0.001** | 0.94 | 0.93 – 0.96 | **<0.001** |
| **eGFR** | 0.99 | 0.98 – 1.00 | 0.067 | 0.99 | 0.98 – 1.00 | **0.031** | 0.99 | 0.98 – 1.00 | **0.013** | 0.99 | 0.98 – 0.99 | **<0.001** | 0.99 | 0.98 – 0.99 | **<0.001** |
| **HTN [Yes]** | 0.71 | 0.38 – 1.33 | 0.282 | 0.84 | 0.49 – 1.45 | 0.538 | 0.81 | 0.49 – 1.32 | 0.391 | 0.92 | 0.59 – 1.43 | 0.706 | 1.01 | 0.66 – 1.53 | 0.964 |
| **DM [Yes]** | 1.82 | 1.05 – 3.14 | **0.033** | 1.67 | 1.05 – 2.66 | **0.031** | 1.7 | 1.09 – 2.65 | **0.019** | 1.28 | 0.86 – 1.89 | 0.222 | 1.33 | 0.92 – 1.93 | 0.129 |
| **NLR** | 1.04 | 1.01 – 1.08 | **0.009** | 1.04 | 1.01 – 1.07 | **0.007** | 1.04 | 1.01 – 1.07 | **0.008** | 1.03 | 1.01 – 1.06 | **0.018** | 1.03 | 1.01 – 1.06 | **0.008** |
| **Troponin** | 1.46 | 0.54 – 3.98 | 0.458 | 1.38 | 0.52 – 3.65 | 0.518 | 1.53 | 0.51 – 4.59 | 0.446 | 1.99 | 0.66 – 6.04 | 0.223 |  |  |  |
| **LDH** | 1 | 1.00 – 1.00 | **0.037** | 1 | 1.00 – 1.00 | **<0.001** | 1 | 1.00 – 1.00 | **<0.001** |  |  |  |  |  |  |
| **ABO [AB]** | 1.09 | 0.32 – 3.73 | 0.892 | 1.03 | 0.32 – 3.31 | 0.955 |  |  |  |  |  |  |  |  |  |
| **ABO [B]** | 0.62 | 0.29 – 1.35 | 0.228 | 0.82 | 0.43 – 1.56 | 0.544 |  |  |  |  |  |  |  |  |  |
| **ABO [O]** | 0.48 | 0.27 – 0.87 | **0.015** | 0.56 | 0.34 – 0.94 | **0.027** |  |  |  |  |  |  |  |  |  |
| **Ddimer** | 1.07 | 1.02 – 1.12 | **0.005** |  |  |  |  |  |  |  |  |  |  |  |  |
| **Observations** | 529 | | | 656 | | | 791 | | | 881 | | | 974 | | |
| **R^2^ Tjur** | 0.434 | | | 0.419 | | | 0.422 | | | 0.376 | | | 0.367 | | |
| **AIC** | 414.49 | | | 533.21 | | | 596.58 | | | 720.259 | | | 798.314 | | |
