# Supplementary material for: SARS-CoV-2 PCR cycle threshold at hospital admission associated with patient mortality
Source: PLoS One. 2020 Dec 31;15(12):e0244777. doi: 10.1371/journal.pone.0244777 (PMC7774957; doi:10.1371/journal.pone.0244777)
Supplement: S3 Table — (DOCX) [file pone.0244777.s004.docx]

**S3 Table.** Sensitivity Analysis: examining the effect of covariate selection on study findings.

|  | **Model 6** | | | **Model 7** | | | | | **Model 8** | | | | | | | **Model 9** | | | | | | | **Model 10** | | | | | | |
| --- | --- | --- | --- | --- | --- | --- | --- | --- | --- | --- | --- | --- | --- | --- | --- | --- | --- | --- | --- | --- | --- | --- | --- | --- | --- | --- | --- | --- | --- |
| ***Predictors*** | ***OR*** | ***CI*** | ***p*** | ***OR*** | | ***CI*** | | ***p*** | ***OR*** | | | ***CI*** | | | ***p*** | ***OR*** | ***CI*** | | | ***p*** | | | ***OR*** | ***CI*** | | | ***p*** | | |
| **Cycle Threshold** | 0.91 | 0.89 – 0.94 | **<0.001** | 0.91 | | 0.89 – 0.94 | | **<0.001** | 0.92 | | | 0.89 – 0.95 | | | **<0.001** | 0.91 | 0.89 – 0.94 | | | **<0.001** | | | 0.91 | 0.88 – 0.93 | | | **<0.001** | | |
| **Age** | 1.03 | 1.02 – 1.05 | **<0.001** | 1.03 | | 1.01 – 1.04 | | **<0.001** | 1.03 | | | 1.02 – 1.05 | | | **<0.001** | 1.04 | 1.03 – 1.05 | | | **<0.001** | | | 1.05 | 1.04 – 1.06 | | | **<0.001** | | |
| **Gender [M]** | 2.01 | 1.39 – 2.91 | **<0.001** | 2.01 | | 1.39 – 2.90 | | **<0.001** | 2.01 | | | 1.40 – 2.90 | | | **<0.001** | 2.1 | 1.47 – 3.01 | | | **<0.001** | | | 1.79 | 1.28 – 2.48 | | | **0.001** | | |
| **BMI** | 1.02 | 0.99 – 1.05 | 0.141 | 1.02 | | 0.99 – 1.05 | | 0.123 | 1.02 | | | 0.99 – 1.05 | | | 0.121 | 1.02 | 1.00 – 1.05 | | | 0.114 | | | 1.02 | 1.00 – 1.04 | | | 0.072 | | |
| **Pulse Ox** | 0.89 | 0.87 – 0.92 | **<0.001** | 0.89 | | 0.87 – 0.92 | | **<0.001** | 0.89 | | | 0.87 – 0.92 | | | **<0.001** | 0.89 | 0.87 – 0.92 | | | **<0.001** | | | 0.89 | 0.87 – 0.92 | | | **<0.001** | | |
| **DBP** | 0.94 | 0.93 – 0.96 | **<0.001** | 0.94 | | 0.93 – 0.96 | | **<0.001** | 0.94 | | | 0.93 – 0.95 | | | **<0.001** | 0.94 | 0.93 – 0.95 | | | **<0.001** | | |  |  | | |  | | |
| **eGFR** | 0.99 | 0.98 – 0.99 | **<0.001** | 0.99 | | 0.98 – 0.99 | | **<0.001** | 0.99 | | | 0.98 – 0.99 | | | **<0.001** |  |  | | |  | | |  |  | | |  | | |
| **HTN [Yes]** | 0.99 | 0.66 – 1.50 | 0.971 | 1.07 | | 0.72 – 1.60 | | 0.736 |  |  | | |  | | |  | |  | | |  | |  | |  | | |  | |
| **DM [Yes]** | 1.31 | 0.91 – 1.90 | 0.146 |  |  | |  | |  | |  | | |  | |  | | |  | | |  |  | | |  | | |  |
| **Observations** | 976 | | | 976 | | | | | 1006 | | | | | | | 1040 | | | | | | | 1041 | | | | | | |
| **R^2^ Tjur** | 0.36 | | | 0.358 | | | | | 0.355 | | | | | | | 0.341 | | | | | | | 0.225 | | | | | | |
| **AIC** | 808.416 | | | 808.527 | | | | | 825.485 | | | | | | | 857.24 | | | | | | | 968.337 | | | | | | |
